# The first evaluation of the effectiveness of canine vaccination schedule by two commercial vaccines in Iran

**DOI:** 10.1186/s12917-022-03219-2

**Published:** 2022-03-29

**Authors:** F. Shams, H. Pourtaghi, Z. Abdolmaleki

**Affiliations:** 1 Department of Clinical Sciences, Karaj Branch, Islamic Azad University, Karaj, Iran; 2 Department of Microbiology, Karaj Branch, Islamic Azad University, Karaj, Iran; 3 Department of Pharmacology, Karaj Branch, Islamic Azad University, Karaj, Iran

**Keywords:** Canine parvovirus type 2, Vaccine, Puppies, Maternal derived antibodies

## Abstract

**Background:**

Canine Parvovirus type 2 (CPV-2) is a member of the Parvoviridae family with a global distribution and causes pathogenicity in puppies aged from 6 weeks to 6 months. It should be noted that Maternally Derived Antibodies (MDA) have protection against CPV-2 in the first weeks of puppies’ life. However, MDA declines with age. The most important influential factor is timely vaccination against CPV-2.

**Methods:**

In this study, 24 healthy 8-week-old terrier puppies were selected and divided into three identical groups based on a randomized, double-blind comparative trial. One of which was called the control group that was injected with the physiological serum. The second group was the group A that was vaccinated by the vaccine provided by Biocan DHPPi+L (Bioveta, Czech). The third group was group B that was vaccinated by the vaccine of Duramune Max 5 + LCI / GP (Fort Dodge Animal Health, USA) from 8 to 16 weeks of their life at every 4 weeks. Then serum samples were analyzed with HI and ELISA tests.

**Results:**

The MDA titer was protective in some puppies until 18 weeks of age. Also, after the first vaccination, all puppies had a protective titer against CPV-2, and Duramune vaccine had seroconverted after the first injection and Biocan had seroconverted after the second injection.

**Conclusions:**

It is recommended that to reduce the risk of vaccine failure: such as the MDA titer should be measured in puppies before designing a vaccination schedule.

## Background

Canine parvovirus type 2 (CPV-2) is a member of the Parvoviridae family and Protoparvovirus genus [1]. CPV-2 is a non-envelope DNA virus that has a high resistance in the environment [2]. The genome of CPV-2 is a single-stranded DNA that encodes with two nonstructural proteins (NS-1 and NS-2) and three structural proteins (VP1, VP2 and VP3) [3]. CPV-2 is a highly infectious virus that causes morbidity and mortality in the puppies [4]. Moreover, parvovirus is found in other mammals such as foxes, wolves, cats and skunks [5]. Parvovirus can infect dogs at any age but it is more common between 6 weeks to 6 months after the birth [6]. It has clinical signs such as lethargy, mucoid to hemorrhagic diarrhea, vomiting, fever, anorexia, depression and sometimes leukopenia and heart failure [7].

It is known that prevention of infectious diseases needs preventive treatment. Therefore the most essential way to prevent parvovirus disease is timely and effective vaccination [8]. The vaccine stimulates the cellular and humoral immune system to produce antibodies [9]. However, it should be noted that age plays a crucial role in the number of booster vaccines [10]. The Maternal Derived Antibody (MDA) titer decreases by passing the time that causes to reduce the interference by the vaccine [11]. Thus, it is necessary to measure the MDA titer and design a vaccination schedule to reduce the cost of vaccine failure. The world small animal veterinary association (WSAVA) group has a guideline for vaccination which, based on, the best time for the first vaccination in puppies were 6–9 weeks from the birth, and then repeated every 3–4 weeks. The last vaccine should be in accordance with 16 weeks after the birth or more [12]. Although there is not enough study about the effect of MDA on vaccines or evaluation of the effectiveness of this guideline. Currently, there are reported works in the literature which proved that MLV has a more effective response than inactivated vaccine [13]. However, choosing between MLV vaccine brands has always been challenging because they have different immunization levels [13]. Thus, comparing between immunization responses with the two most used vaccines will help have a good choice.

The objectives of this study were in the followings; i) the evaluation of changing MDA titer in the passing of time; ii), evaluation of the efficacy for the vaccination schedule in Iran based on WSAVA vaccination guidelines; and iii) evaluation of the possible effect of MDA on vaccines and efficacy of two commercial vaccines against CPV-2 which occurred for the first time in Iran.

## Methods

### Study design

This study is based on a randomized, double-blind comparative trial. All studies were approved by the Iran national Ethics Committee on the Iran Islamic Azad University, Karaj, Research Ethics Committee (code: IR.IAU.K.REC.1400.16).

### Animals design

In the current study, twenty-four (12 males and 12 females), healthy, terrier puppies were selected from two growing farms with the appraently healthy dogs or no positive results in the ELISA test for canine parvovirus in the last 2 years. Appropriate permission was obtained from the farms owners to use terrier puppies for the study.

Dams of all puppies had a vaccination schedule against CPV-2, and the last vaccination was 5 months prior to the delivery. Before entry to the study, all puppies were physically examined by one of faculty member of Karaj Islamic Azad University Dr. Ali Taghipour, and got cell blood count to make sure that no abnormality and disease could interfere with the results. Then, they received anti helminth drugs twice at three and 6 weeks of age. Fenbendazole had orally administrated every 24 h for 3 days each time. Puppies were weaned at 8 weeks of age. Afterward, they were randomly divided into three groups and coded by an impartial vet who was unaware of the trial for maintaining the blinding of the study. The team of researchers in this study had no contact with puppies and vaccines until the vaccination day. Puppies in these groups were kept in three geographically separate regions. They were housed in wire cages. The puppies were fed with commercial dog food that was formulated to grow them. This diet was made three times a day, and at the same time, the puppies had free access to sanitize water.

### Sampling design and vaccines

The puppies have been vaccinated in 4 weeks intervals at weeks 8, 12, and 16 of their life.

- Control Group: Injected with 1 ml normal saline
- Group A: Vaccinated with Biocan DHPPi+L Bioveta, Czech
- Group B: Vaccinated with Duramune Max 5 + LCI/GP; Fort Dodge Animal Health, USA

The vaccines and normal saline were injected subcutaneously in the dorsal region of the shoulder or neck. Blood samples were collected 2 weeks after each injection. About 5 ml of blood was collected into a plain tube and allowed to clot. The serum was removed and frozen.

### Laboratory evaluation

For the ELISA test, all blood samples were analyzed in the serology laboratory of the veterinary faculty of Karaj Islamic Azad University (KIAU). The MDA and post-vaccination response of antibody titer against CPV-2 were evaluated by using an indirect ELISA kit (Parvo Ab ELISA 96, Ingenasa). All procedures and stages were carefully followed to satisfy the manufacture’s recommendation.

For Haemagglutination Inhibition (HI) test first, 25 μl of PBS was added to all wells. Then, 25 μl of dog serum was added to well number one and the pipetting was performed. 25 μl of the contents of the first well were also transferred to the second and pipetting was performed again. Similarly, 25 μl of the contents of the previous well was transferred to the next well, (Serial dilutions was started from 1:10). Then 25 μl of 8 HA virus was added to all wells. Incubation with the possible antibodies was performed at room temperature for 60 min to neutralize the virus. Finally, 50 μl of 1% cat RBC was added to all wells and the microplate was incubated at 4 °C for 12 h. The headline was equivalent to the last well in which hemagglutination of the virus was prevented and a button was observed inside [12].

### Statistical analysis

SAS 9.2 software was used as a strong statistical tool to analyze the obtained data. We used the Bartlett test to control the variance independence. The ANOVA and Fisher test were also applied to check changes in antibody titer against CPV-2 in the three groups of the subject of this study in four times. The mean value of antibodies against the CPV-2 in each group was compared with Duncan’s multiple range test. It should be noted that confidence intervals of 5 and 1%, were evaluated for each analysis. Chi-Squared was used for analyzing the HI data with a *P*-value of 0.01%.

## Results

At the beginning of the study, the mean of the MDA titer in the three groups were not significantly different. However, after the first vaccination, groups A and B showed asignificantly different MDA compared to the control groups, i.e. *P* > 0.01.

### Evaluation of changing MDA titer in the passing of time against CPV-2

HI results show that before the study, all puppies in the control group were in the safe titer ≥1:80, and also ELISA results showed the mean of titer was 1.08 ± 0.34 (Table 1).

**Table 1:** Pups in control group showing HI titer against CPV-2 at different ages

	1:20	1:40	1:80	1:160	1:320	1:640	1:1280
**Week 8**	–	–	4	4	–	–	–
**Week10**	–	1	5	2	–	–	–
**Week14**	–	4	3	1	–	–	–
**Week18**	–	6	1	1	–	–	–

In the 10 weeks of age, only 12.5% of puppies reached 1:40 HI titer that is not immune level. Also, ELISA data showed that the mean titer was (0.89 ± 0.34), confirming that this decrease is not significantly different from the titer of the beginning.

At the weeks of 14, half of the puppies were safe and the next half had 1:40 HI titers and also the ELISA titer was (0.71 ± 0.32). This decrease was not significantly different from the 10-week-age, but it was significantly different from the beginning (*P* > 0.01).

**Fig. 1:**
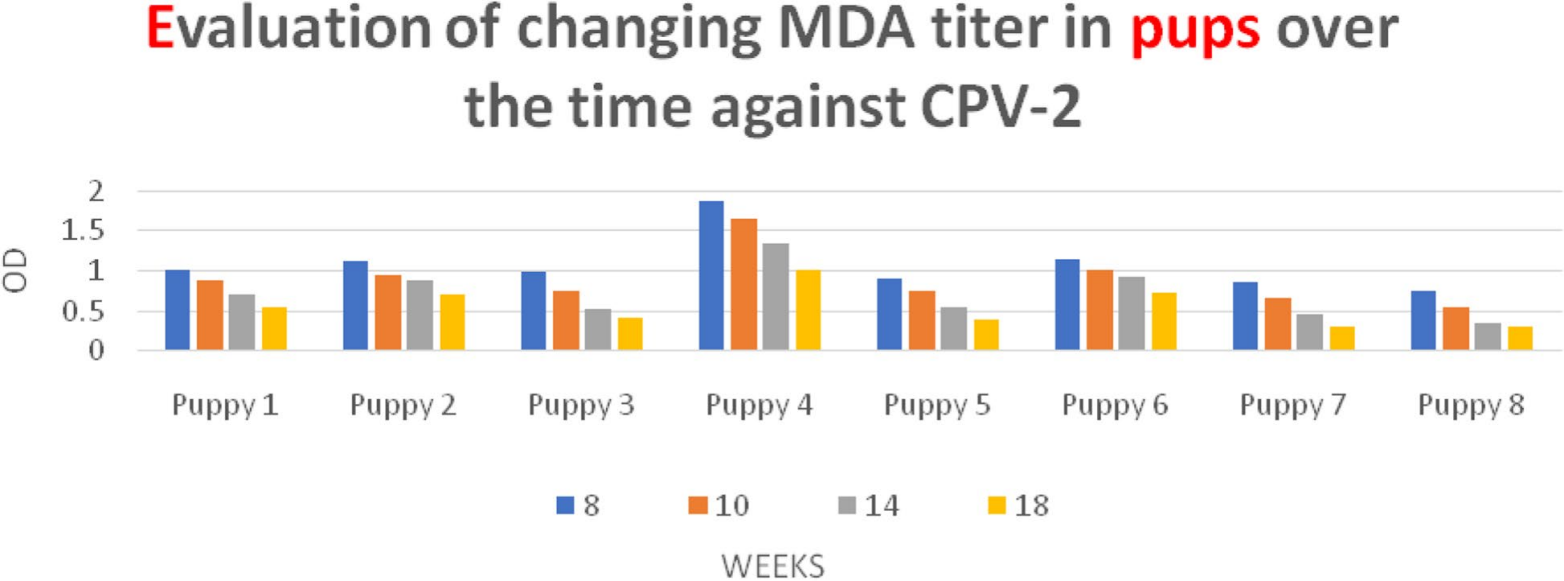
The changing trends in antibody titers in ELISA against CPV-2 in the control group (unvaccinated group)

At the weeks of 18, 75% of the puppies had 1:40 HI titers, 12.5% had 1:80 and also 12.5% had 1:160 from the beginning of the study. ELISA titer was (0.55 ± 0.24). This reduction was not significantly different from the results at weeks of 10 and 14; however, it showed a significantly different outcome as compared to the data from the beginning (*P* > 0.01) (Fig. 1).

### Evaluation of the possible effect of MDA on vaccines against CPV-2 and efficacy of two commercial vaccines

At the week of 8, the mean of MDA titer for the group A was 1.24 ± 0.46 and the group B was 1.13 ± 0.19. There was no a significant difference between these two groups. Besides, HI results have shown that all puppies had an immune level between 1:80 to 1:160 (Table 2).

**Table 2:** Pups in vaccinated groups showing HI titer against CPV-2 at different ages

	1:20	1:40	1:80	1:160	1:320	1:640	1:1280	1:2560
**WEEK 8**								
A	–	–	4	4	–	–	–	–
B	–	–	2	6	–	–	–	–
**WEEK10**								
A	–	–	–	8	–	–	–	–
B	–	–	–	2	–	6	–	–
**WEEK14**								
A	–	–	–	1	1	3	3	–
B	–	–	–	–	–	4	4	–
**WEEK18**								
A	–	–	–	1	–	1	6	–
B	–	–	–	–	–	1	7	–

At 10 weeks of age, the mean antibody titer for group A was 1.64 ± 0.22 and for the, group B was 2.21 ± 0.56. There was a significant difference between the two groups (*P* > 0.01). Furthermore, the results showed that half of the puppies in the group A have increased one log in their HI titer (1:160) and most of the puppies in the group B had an increased 2–3 log in HI titer (1:160–1:640).

At 14 weeks of age, the mean MDA titer for the group A and group B was 2.6 ± 0.72 and 2.72 ± 0.13, respectively. There was no statistically significant difference between the two groups. In addition, most of the puppies in the group A had an increased 2–3 log in their HI (1:160–1:1280) and half of the puppies in the group B had an increased onelog in HI titer (1:640–1:1280).

**Fig. 2:**
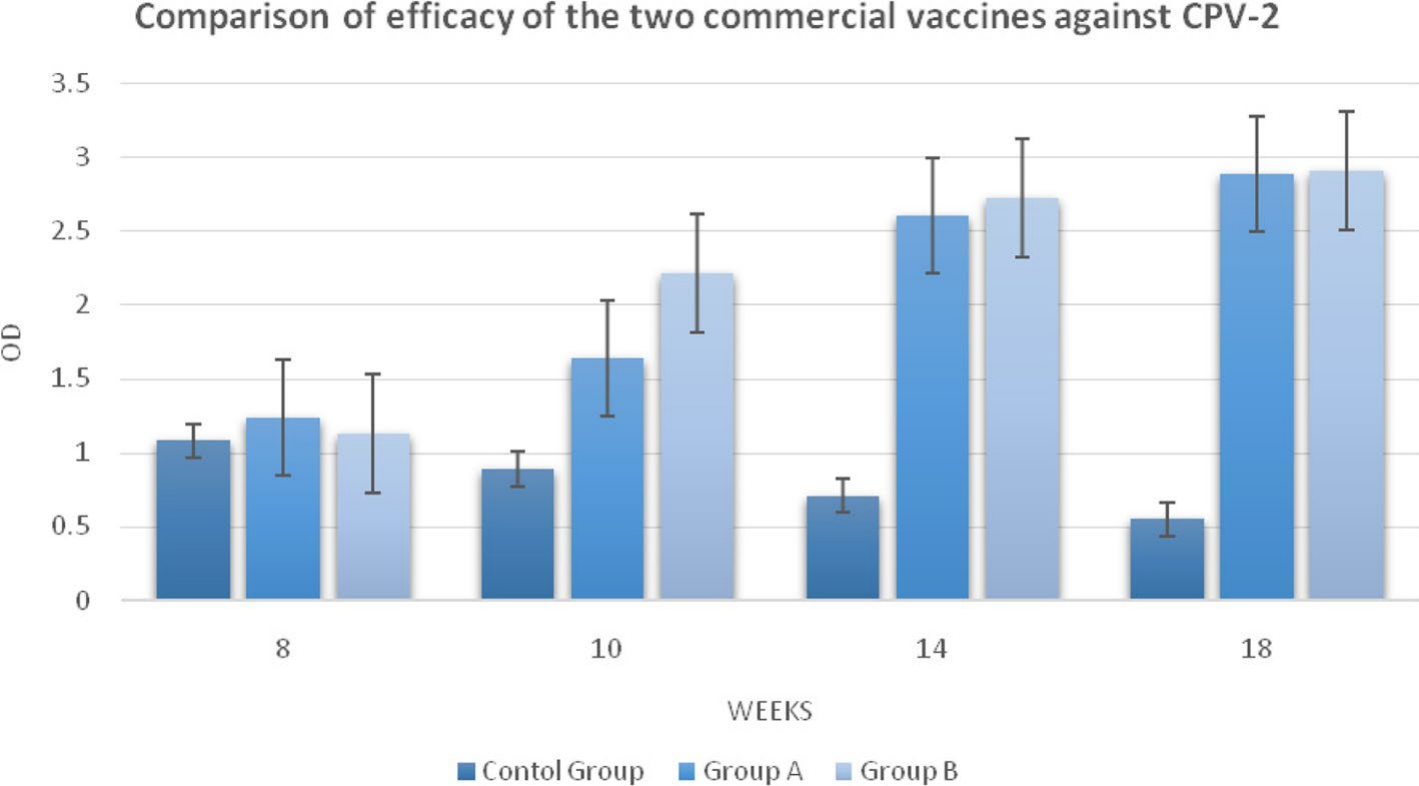
Comparison of the antibody titer in ELISA of the two commercial vaccines against CPV-2 during 3 doses of vaccination

At 18 weeks of age, the mean MDA titer for group A and group B was 2.89 ± 0.55 and, 2.91 ± 0.17, respectively. Again like the results in the 14 weeks, there was no statistically significant difference between the two groups. Note that half of the puppies in the group A had an increased one log HI (1:160–1:1280). However, some of the puppies in the group B had an increased one log titer (1:640–1:1280) (Fig. 2).

## Discussion

Vaccination is currently recognized as the most effective solution for the control and prevention of most infectious diseases [14]. In this regard, the usefulness of vaccine in this disease prevention has been considered in a study while only a few hours after vaccination, the dogs were in a contaminated environment and proved the vaccine effectiveness [15]. In the another study, it was shown that the puppies were protective after 3 days of vaccination against the parvovirus challenge [16]. It should be noted, that this study was performed in accordance with the K.D.Altman study, in which sampling was done 2 weeks after vaccination as that is the best time for the immune response to the parvovirus vaccine [17].

According to the previous studies, the live attenuated vaccines have a higher ability to stimulate the immune system than the inactivated vaccines [18]. Which, It is recommended for puppies with high MDA [19]. Moreover, the recent studies have shown that new MLV vaccines have been discovered that can be used in pregnant bitch or puppies under 6 weeks of age [20]. However, the use past of inactivated vaccines in immunization during pregnancy and exotic animals should not be ignored [21].

Despite, all the emphasis on receiving the core vaccines, the statistics of CPV-2 disease are still high, especially in developing countries [22]. That may be attributed to the vaccine failure. Thus, a good way to prevent this problem is to perform an antibody titer test, which the HI test is the standard gold test for detection of parvovirus antibodies [23]. Besides, the antibody testing could be performed at least 1 month after the last vaccination administered at 16 weeks or more and can be repeated every 3 years in the case of a positive result [17]. Besides, there are several factors that affect on vaccine response including high MDA titer, vaccine storage conditions and failure to maintain a cold chain [24], the presence of parasitic diseases that can reduce nutrition then causing immunosuppression and stress [25]. Thus in this study, all puppies have treated with anthelminthic drugs twice before the beginning of the study, and the most important factor is MDA, which can interfere with modified live virus and cause vaccine failure [26]. Generally, MDA is known as the first defense against pathogens in puppies. Based on the previous studies, a relationship has been observed between the absorbed MDA and the duration of protection, which is proof of the importance of colostrum in newborn puppies [24]. Despite this, MDA has decreased over time and most studies have reported that the MDA protective titer exists until 13–15 weeks of age [27]. In this regard, some solutions can be used to nullify the MDA interference such as determining the MDA titer, alternative of injective vaccines in the young puppies, use high-titer vaccines in areas with high prevalence [15]. In this regard, these vaccines are 2 to 3 logs higher than other vaccines, which the MLV of the vaccines are not neutralized with the presence of moderate MDA titers [16]. Morover, an alternative to injecting vaccines including inhaled or oral vaccines, have been suggested however, do not work well [28]. The best time to determine the MDA titer is in 4 to 6 weeks. Considering the downward slope of the antibody titer and having a half-life of 10 days, the appropriate vaccination time by using HI test [15] can be predicted. In this study, we used this solution to prevent MDA interference, which in our control group 25% of the puppies at 18 weeks of age were also able to maintain their maternal level of immunity against CPV-2. These results are matched with the study conducted by L.J.Larson et al. [29]. They reported that some puppies could not gain active immunity with vaccines until at least 18 weeks of age due to high MDA titers, so in these cases it is better to prescribe several doses of vaccine [29].

Based on WSAVA guidelines, the dogs that have 8 week-age should be vaccinated every 3–4 weeks until they reached to the 16 weeks of life or more [30]. In Iran, the vaccination program is started at 8 weeks for puppies, during which they receive 3 vaccination periods every 4 weeks. However, to our best knowledge, no study has been conducted in Iran to evaluate the effectiveness of this schedule. The results of this study have proved that the current vaccination schedule can be used by veterinarians of Iran. After the first vaccination in the group A, two of the puppies have decreased the titers. In the group B, one of the puppies have decreased the titer and one of them had no change in her titer, however, all puppies have been protected after the first vaccination. Thus, the vaccination could be stopped at this stage. In the second vaccination, despite all the puppies in both groups had the protective titers, one of the puppies in group A had decreased titer. At this stage, the protective titers from vaccination had increased, and in the third vaccination, the results were similar to the second vaccination and increased the protective titer. In another study that was performed on the dogs, it is shown that after 2 weeks of vaccination, in the first dose 98% and the second dose 100% of puppies were protected against CPV-2 [31].

In the current study, and in the first vaccination, the group B had seroconverted and in the second vaccination, the group A had seroconverted. Both groups had performed the same in the third vaccination. Thus, vaccine B can be used to achieve faster the immune titer. These results were found in agreement with the study conducted by Coyne [32].

## Conclusion

Despite of providing the global vaccination schedule, parvovirus is still prevalent and causes the death of a large number of young dogs annually. Currently, the most effective way to combat this virus is an effective vaccination. In this regard, the vaccine should not interfere with the MDA. Thus, it is necessary to measure the level of MDA titer in puppies less than 16 weeks old before starting vaccination activities to design the best vaccination schedule. However, WSAVA guidelines can be a suitable alternative in the absence of facilities. Besides in an emergency situation can be used Duramune Max 5 vaccine to achieve faster the immune titer.

## Data Availability

The datasets used and/or analyzed during the current study are available from the corresponding author on reasonable request.
